# Coexistence of *Candida* species and bacteria in patients with cystic fibrosis

**DOI:** 10.1007/s10096-019-03493-3

**Published:** 2019-02-09

**Authors:** Johanna Haiko, Baharak Saeedi, Gabriella Bagger, Ferenc Karpati, Volkan Özenci

**Affiliations:** 1 Department of Clinical Microbiology, Karolinska University Hospital, Stockholm, Sweden; 2 Stockholm CF-Center, Department of Pediatrics, Karolinska University Hospital, Huddinge, Stockholm, Sweden; 3 Division of Clinical Microbiology F 72, Department of Laboratory Medicine, Karolinska Institutet, Karolinska University Hospital, Huddinge, SE-141 86 Stockholm, Sweden

**Keywords:** Coexistence of yeast and bacteria, *Candida* spp., Cystic fibrosis

## Abstract

Cystic fibrosis (CF) patients become colonized by pathogenic bacteria as well as by *Candida* species. The interplay between different microorganisms may play a key role in the prognosis of CF. The aim of the study was to analyze the coexistence patterns of bacteria and *Candida* spp. in sputum samples of patients with CF and to compare these patterns with the results of patients with other respiratory disorders (ORD). Sputum samples from 130 patients with CF and 186 patients with ORD were cultured on six different agar plates promoting the growth of bacteria and yeasts. Bacterial and *Candida* species were identified with MALDI-TOF MS. Pathogenic bacteria were found in 69.2% of the sputum samples of the CF patients, and in 44.1% the patients with ORD. CF patients tended to have growth of *Pseudomonas aeruginosa* and *Staphylococcus aureus* in sputum more often than patients with ORD. Overall, there was no difference in the coexistence of pathogenic bacteria and *Candida* spp. in these patient groups. However, when analyzed at the species level, *P. aeruginosa* and *S. aureus* coexisted with *Candida* spp. more frequently in sputum samples of CF patients compared with patients with ORD. Also, when analyzed according to age, it was shown that the adult (≥ 18 years) CF patients had a higher rate of coexistence of any pathogenic bacteria and *Candida* spp. than the children with CF and the adult patients with ORD. The rate for colonization with *Candida* together with pathogenic bacteria is increased in adult patients with CF.

## Introduction

Cystic fibrosis (CF) is the most common monogenetic autosomal recessive disease in Northern Europe. The disease is caused by mutations in a cystic fibrosis transmembrane conductance regulator (CFTR) gene whose product is a chloride channel (Williams et al. [1]). CFTR mutations result in reduced excretion of chloride ions in the apical membrane of epithelial cells. Consequently, water absorption and viscid secretions increase, leading to defective mucociliary clearance. The disease affects mainly the epithelial cells in the intestine, respiratory system, pancreas, gall bladder, and sweat glands. Excessive mucus production and defected mucociliary clearance result in obstructive lung disease and chronic bacterial infections leading to respiratory failure, which is still the major cause of morbidity and mortality [2]. The colonization of the lower respiratory tract with microorganisms progresses with the duration of cystic fibrosis. In the early phases, the most common bacteria are *Staphylococcus aureus* and *Haemophilus influenzae*, whereas a wide range of bacteria, mainly *Pseudomonas aeruginosa*, *Burkholderia cepacia* complex, and other Gram-negative bacteria as well as fungal species, are isolated in the later stages of the disease [1]. There are several studies analyzing the prevalence of the pathogenic bacteria and fungi in patients with CF (for recent reviews, see [3–5]).

There is accumulating knowledge on the interplay between bacteria and yeasts. Recent studies have showed that the interaction between *P. aeruginosa* and *Candida* spp. can result in significant changes in the pathogenicity of *P. aeruginosa* [6–10]. Since *P. aeruginosa* is regarded as one of the most important pathogens in CF, detection and isolation of this pathogen are routinely performed. In contrast, *Candida* spp. are generally considered as colonizers of the lower respiratory tract and regarded as not clinically relevant. Therefore, detection and identification of yeasts are performed occasionally.

There is limited data on the coexistence of viable bacteria and yeasts in the respiratory tract of CF patients [11, 12]. In addition, hitherto published studies have focused on describing the presence of yeasts and bacteria solely in patients with CF without comparing the data with patients with other respiratory diseases.

The aim of the present study was to document the coexistence of *Candida* spp. and pathogenic bacteria in the lower respiratory tract of the patients with CF and to compare these patterns with the patients with other respiratory disorders.

## Materials and methods

### Sputum samples

Clinical sputum samples used in this study were obtained from the patients with CF who are followed at the Stockholm CF Center at Karolinska University Hospital, Huddinge. Samples from the patients with other respiratory disorders were received mainly from the Lung and Allergy Clinic and rarely from Infectious Diseases Clinic for investigation for bacterial culture. The samples were cultured and processed at the Department of Clinical Microbiology, Karolinska University Hospital, Huddinge, Sweden, during a 9-week period in 2017.

### Culturing of sputum samples

After sputolysin treatment, the sputum samples were inoculated on the following agar plates to identify the growth of pathogenic bacteria: chocolate agar, blood with crystal violet, cystine–lactose–electrolyte-deficient agar, *Pseudomonas* agar, and *Burkholderia cepacia* agar. The plates were incubated for 2 days at 35 °C in ambient air with the exception of the blood with crystal violet plates which were incubated in 5% CO_2_. The agar plates mentioned above are standard media in the detection of pathogenic bacteria from clinical lower respiratory tract samples in our laboratory. To detect the growth of yeasts in the study material, the samples were also inoculated onto two selective agars to promote the growth of *Candida* spp.: Sabouraud dextrose (Becton Dickinson and Company) and chromogenic yeast agar (CHROMagar), which were incubated at 35 °C in ambient air for 5 days.

### Identification of microorganisms

The colony morphology and the growth characteristics of the microorganisms that were detected on agar plates were visually evaluated. The bacterial colonies that were interpreted as clinically relevant were analyzed by MALDI-TOF MS. In addition, all the colonies that were grown on Sabouraud dextrose and CHROMagar were analyzed with MALDI-TOF MS for the identification of *Candida* spp. MALDI-TOF MS (Bruker Daltonics) analysis was performed according to the manufacturer’s instructions with the use of alpha-cyano-4-hydroxycinnamic acid (HCCA) matrix. Yeasts that did not give a valid result, i.e., with a score < 1.7, were treated with full ethanol-formic acid-acetonitrile extraction protocol and reanalyzed by MALDI-TOF MS.

Bacterial species that were regarded as clinically significant were *P. aeruginosa* and other *Pseudomonas* spp., *S. aureus*, *H. influenzae*, Enterobacteriaceae, *Stenotrophomonas maltophilia*, *Streptococcus pneumoniae*, *Moraxella catarrhalis*, *Achromobacter xylosoxidans*, *Burkholderia cepacia* complex, *S. pyogenes*, and *Neisseria meningitidis*.

### Statistical methods

The presence of individual microorganisms and coexistence of bacteria and *Candida* spp. in adult CF patients, children with CF, and patients with other respiratory disorders were compared using the chi-square test. Fisher’s exact test was used when two individual patient groups were compared due to a lower sample size. The GraphPad Prism version 5.01 for Windows, GraphPad Software, San Diego, CA, USA (www.graphpad.com), was used for analysis. Values of *p* < 0.05 were statistically significant.

## Results

In total, clinical sputum samples from 130 patients with CF and 186 patients with other respiratory disorders were included in the study. Only one sample per patient was analyzed. The patient characteristics are summarized in Table 1. The mean age of the CF patients and patients with other respiratory disorders were 25.9 and 59.0 years, respectively. In the CF group, 52/130 (40.0%) patients were children (< 18). The data from children with CF and adult patients were also analyzed separately. In patients with other respiratory disorders, there were only 9/186 (4.8%) children. Due to the very low prevalence of children in patients with other respiratory disorders and no significant difference in the presence of different microorganisms in children and adults, the data were analyzed for all patients in this group. The proportion of males was 50.0% in the CF patients and 39.8% in the patients with other respiratory disorders.

**Table 1:** The characteristics of the patients with cystic fibrosis (CF) and other respiratory disorders included in the study

	Cystic fibrosis (*n* = 130)	Other respiratory disorders (*n* = 186)
Gender		
Male	50.0%	39.8%
Female	50.0%	60.2%
Age		
≥ 18 years	60.0%	95.2%
< 18 years	40.0%	4.8%
Mean	25.9 years	59.0 years
Median	16.0 years	60.0 years

### Growth of pathogenic bacteria

**Fig. 1:**
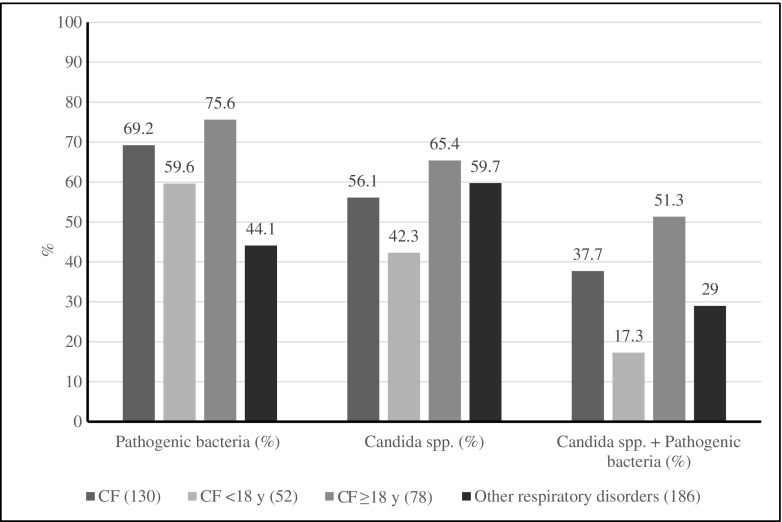
Detection of pathogenic bacteria, *Candida* spp., and coexistence of pathogenic bacteria and *Candida* spp. in sputum samples in patients with cystic fibrosis (CF) and other respiratory disorders. Pathogenic bacteria: *Pseudomonas aeruginosa*, *Staphylococcus aureus*, *Haemophilus influenzae*, Enterobacteriaceae, *Stenotrophomonas maltophilia*, *Streptococcus pneumoniae*, *Moraxella catarrhalis*, *Achromobacter xylosoxidans*, *Burkholderia cepacia* complex, *Streptococcus pyogenes*, *Neisseria meningitidis*, and *Pseudomonas* species

Pathogenic bacteria, e.g., *P. aeruginosa* and other *Pseudomonas* species, *S. aureus*, *H. influenzae*, Enterobacteriaceae, *S. maltophilia*, *S. pneumoniae*, *M. catarrhalis*, *A. xylosoxidans*, *B. cepacia* complex, *S. pyogenes*, or *N. meningitidis*, were detected in 90/130 (69.2%) of the sputum samples of the CF patients, and in 82/186 (44.1%) of the samples of the patients with other respiratory disorders (*p* < 0.0001) (Fig. 1). When the patients with CF were analyzed according to age, there was no difference in the detection of pathogenic bacteria between adult (≥ 18 years) CF patients and children (< 18 years) with CF, 59/78 (75.6%) and 31/52 (59.6%), respectively (*p* > 0.05, not significant). The adult CF patients had higher levels of pathogenic bacteria compared with patients with other respiratory disorders, 59/78 (75.6%) and 82/186 (44.1%), respectively (*p* < 0.0001). Children with CF had slightly higher levels of pathogenic bacteria compared with patients with other respiratory disorders, but this difference did not reach statistical significance, 31/52 (59.6%) and 82/186 (44.1%), respectively (*p* = 0.06, not significant) (Fig. 1).

When the three most common pathogenic bacterial species were analyzed, patients with CF had higher numbers of sputum samples with growth of *P. aeruginosa* than patients with other respiratory tract disorders, 51/130 (39.2%) and 32/186 (17.2%), respectively (*p* < 0.0001) (Table 2). The sputum cultures were positive for *S. aureus* in 29.2% of CF patients and only in 8.1% of the patients with other respiratory disorders (*p* < 0.0001). However, no difference was observed in the growth of *H. influenzae* between the two groups (*p* > 0.05, not significant) (Table 2).

**Table 2:** The presence of three pathogenic bacterial species in the sputum samples of patients with CF and other respiratory disorders. Note that one patient may have more than one bacterial species

Patients (*n*)	*P. aeruginosa*, *n* (%)	*S. aureus*, *n* (%)	*H. influenzae*, *n* (%)
CF (130)	51 (39.2)	38 (29.2)	13 (10.0)
CF < 18 years (52)	9 (17.3)	16 (30.8)	6 (11.5)
CF ≥ 18 years (78)	42 (53.8)	22 (28.2)	7 (9.0)
Other respiratory disorders (186)	32 (17.2)	15 (8.1)	21 (11.3)

### Growth of *Candida* spp.

*Candida* spp. were detected in 73/130 (56.1%) CF patients and in 111/186 (59.7%) patients with other respiratory disorders (*p* > 0.05, not significant) (Fig. 1). The adult CF patients had higher levels of *Candida* spp. compared with children with CF, 51/78 (65.4%) and 22/52 (42.3%), respectively (*p* = 0.01). The children with CF had also lower levels of *Candida* spp. compared with other respiratory disorders, 42.3% and 59.7%, respectively (*p* < 0.05) (Fig. 1).

When the detection of *Candida* spp. was analyzed at the species level, we observed that *C. albicans* was the most common *Candida* species in our study material, with 53/130 (40.8%) in CF patient samples and 96/186 (51.6%) in the samples of patients with other respiratory disorders. The next most common *Candida* species were *C. dubliniensis* and *C. glabrata* (Table 3). Other *Candida* spp. were present in less than 20 (6.3%) samples altogether. More than one *Candida* species were detected in only 21/316 (6.6%) of the clinical samples.

**Table 3:** The presence of different *Candida* spp. in the sputum samples of patients with CF and other respiratory disorders. Note that one patient may have more than one *Candida* spp.

Patients (*n*)	*C. albicans*, *n* (%)	*C. dubliniensis*, *n* (%)	*C. glabrata*, *n* (%)
CF (130)	53 (40.8)	18 (13.8)	6 (4.6)
CF < 18 years (52)	14 (26.9)	6 (11.5)	1 (1.9)
CF ≥ 18 years (78)	39 (50.0)	12 (15.4)	5 (6.4)
Other respiratory disorders (186)	96 (51.6)	9 (4.8)	7 (3.8)

### Coexistence of *Candida* spp. and pathogenic bacteria

Overall, in 103/316 (32.6%) clinical sputum samples, growth of both pathogenic bacteria and *Candida* spp. was observed. There was no significant difference between patients with CF and patients with other respiratory disorders in coexistence of *Candida* spp. and pathogenic bacteria, 49/130 (37.7%) and 54/186 (29.0%), respectively (*p* > 0.05, not significant). The adult CF patients had higher levels of coexistence of pathogenic bacteria and *Candida* spp. than patients with other respiratory disorders, 40/78 (51.2%) and 54/186 (29.0%), respectively (*p* < 0.001). The adult CF patients had also significantly higher levels of coexistence of pathogenic bacteria and *Candida* spp. than children with CF, 40/38 (51.3%) and 9/43 (17.3%), respectively (*p* < 0.001). There was no difference between children with CF and patients with other respiratory disorders in the coexistence of *Candida* spp. and pathogenic bacteria (*p* > 0.05, not significant) (Fig. 1).

We also analyzed the coexistence of *Candida* spp. with the three most common bacterial species in our material, i.e., *P. aeruginosa*, *S. aureus*, and *H. influenzae*. This approach revealed that *P. aeruginosa* coexisted with *Candida* spp. more frequently in the sputum samples of CF patients compared with the patients with other respiratory disorders, 29/130 (22.3%) vs. 22/186 (11.8%), respectively (*p* < 0.05; Table 4). Similarly, CF patients had higher coexistence levels of *S. aureus* with *Candida* spp. than the patients with other respiratory disorders, 17/130 (13.1%) vs. 11/186 (5.9%), respectively (*p* < 0.05). However, there was no difference in the levels of coexistence of *H. influenzae* with *Candida* spp. in these two patient groups (*p* > 0.05, not significant).

**Table 4:** Coexistence of *Candida* spp. and the three most common pathogenic bacterial species

Coexisting species	CF (*n* = 130), *n* (%)	Other respiratory disorders (*n* = 186), *n* (%)
*Candida* spp. + *P. aeruginosa*	29 (22.3)	22 (11.8)
*Candida* spp. + *S. aureus*	17 (13.1)	11 (5.9)
*Candida* spp. + *H. influenzae*	5 (3.8)	13 (7.0)

### Growth of only pathogenic bacteria and only *Candida* spp.

**Fig. 2:**
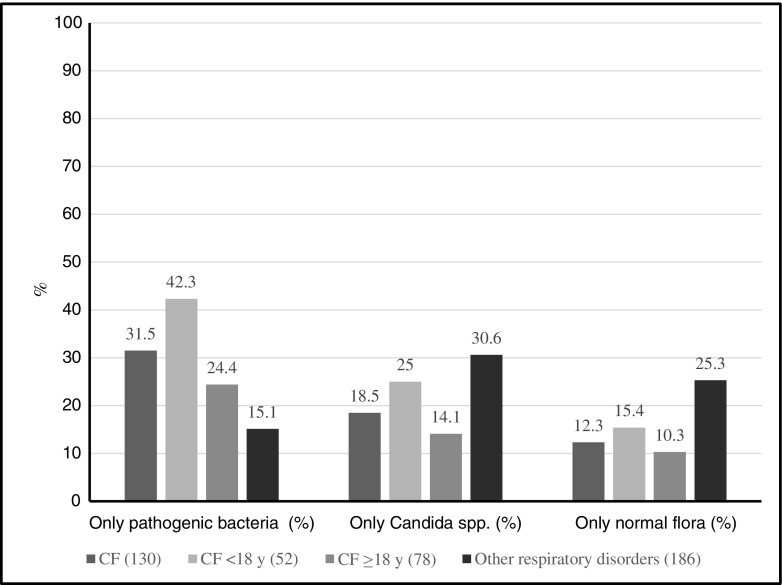
Detection of only pathogenic bacteria, only *Candida* spp., and only normal flora in sputum samples in patients with cystic fibrosis (CF) and other respiratory disorders. Pathogenic bacteria: *Pseudomonas aeruginosa, Staphylococcus aureus*, *Haemophilus influenzae*, Enterobacteriaceae, *Stenotrophomonas maltophilia*, *Streptococcus pneumoniae*, *Moraxella catarrhalis*, *Achromobacter xylosoxidans*, *Burkholderia cepacia* complex, *Streptococcus pyogenes*, *Neisseria meningitidis*, and *Pseudomonas* species

Pathogenic bacteria, in the absence of *Candida* spp., were detected in 69/316 (21.8%) clinical samples included in the study. Patients with CF had higher levels of only pathogenic bacteria (i.e., in the absence of *Candida* spp.) compared with patients with other respiratory disorders, 41/130 (31.5%) and 28/186 (15.0%), respectively (*p* < 0.001). Children with CF had higher levels of growth of only pathogenic bacteria compared with both adult CF patients and patients with other respiratory disorders, 22/52 (42.3%), 19/78 (24.3%), and 28/186 (15.1%), respectively (*p* < 0.05 and *p* < 0.0001, respectively). Adult CF patients and patients with other respiratory disorders had similar levels of growth of only pathogenic bacteria (*p* > 0.05, not significant) (Fig. 2).

Overall, only *Candida* spp. (i.e., in the absence of pathogenic bacteria) were isolated in 81/316 (25.6%) of the clinical samples. Patients with other respiratory disorders had higher levels of only *Candida* spp. compared with patients with CF, 57/186 (30.6%) and 24/130 (18.5%), respectively (*p* < 0.05). Adult CF patients and children with CF had similar levels of growth of only pathogenic *Candida* spp., 11/78 (14.1%) and 13/52 (25.0%), respectively (*p* > 0.05, not significant) (Fig. 2).

## Discussion

Microorganisms tend to exist in multispecies communities in their ecological niches, including the human respiratory tract, and interact with each other in several ways. Hitherto ex vivo studies have shown that these interactions may have both stimulatory and inhibitory effects for the virulence of the microorganisms. It is therefore important to analyze the levels of coexistence of clinically relevant bacteria and yeasts in individual diseases. However, there is limited information on the level of coexistence of the viable microorganisms in chronic diseases such as CF. In the present prospective study, we analyzed the presence of pathogenic bacteria and *Candida* spp. in clinical sputum samples by a culture-based method in patients with CF and patients with other respiratory disorders. Patients with CF were later divided into two groups as children and adults and further analyzed for the coexistence of pathogenic bacteria and *Candida* spp.

One-third (32.6%) of the patients included in the study had both pathogenic bacteria and *Candida* spp. in their lower respiratory tract. The adult CF patients had significantly higher levels of coexistence of pathogenic bacteria and *Candida* spp., both compared with the children with CF and with the patients with other respiratory disorders, 51.2%, 17.3%, and 29.0%, respectively (*p* < 0.001). The underlying reasons for the differences in these three groups in the coexistence of pathogenic bacteria and *Candida* spp. are probably multifactorial and complex.

It is known that the respiratory tract of the CF patients is most often colonized with different *Candida* spp. [11–14]. *Candida* spp. can be regarded as normal respiratory microbiota in moderate amounts and in immunocompetent individuals, but they are considered opportunistic pathogens, particularly in immunocompromised individuals, including CF patients. The role of *Candida* spp. in the pathogenesis of CF is, however, poorly understood. CF patients are increasingly treated with antifungal agents against molds [11], but they more often have long-term antibiotic treatment, and these aspects may explain the prevalence of *Candida* species. The detection of *Candida* spp. in patients with CF might also be related to other factors including the patients’ age and the duration of the disease.

In the present study, when the growth of *Candida* spp. was analyzed, the children with CF had significantly lower levels of *Candida* spp. compared with both adult CF patients and patients with other respiratory disorders, 42.3%, 65.4%, and 59.7%, respectively. Since the levels of *Candida* spp. were similar in adult CF patients and patients with other respiratory disorders, the present data suggest that the colonization of *Candida* spp. might be mainly due to the age of the patient.

When the presence of bacteria was analyzed, we observed that the adult CF patients had significantly higher levels of pathogenic bacteria compared with patients with other respiratory disorders, 75.6% and 44.1%, respectively. The present data suggest that the major difference between adult CF patients and patients with other respiratory disorders in the coexistence of pathogenic bacteria and *Candida* spp. might be due to the abundant presence of pathogenic bacteria in CF patients.

It was previously shown that *P. aeruginosa*, *S. aureus*, and *H. influenzae* are the most common pathogenic bacteria present in the lower respiratory tract of patients with CF [12, 15]. In line with these studies, we observed that CF patients had higher levels of *P. aeruginosa* and *S. aureus* compared with patients with other respiratory disorders, reflecting the importance of these two pathogens in CF.

Detection of *P. aeruginosa* has previously been correlated with the severity of CF [16], as well as with increased inflammation [17]. The existence of *P. aeruginosa* and *C. albicans*, together or alone, in the airways of CF patients, has been associated with worsened outcome of the lung function [18, 19]. Similarly, combinations of *P. aeruginosa* and *A. fumigatus*, *P. aeruginosa* and *S. maltophilia*, and *P. aeruginosa* and *B. cepacia* have been particularly associated with the loss of lung function in CF patients [20]. In the present study, we showed that the CF patients had higher levels of *P. aeruginosa* together with *Candida* spp. compared with patients with other respiratory disorders, 22.3% vs. 11.8%, respectively (*p* < 0.05). The interactions between *C. albicans* and bacteria at the molecular level have been shown to be complex. Chen et al. [8] showed that *C. albicans* produces ethanol, which in turn promotes both the colonization and biofilm formation of *P. aeruginosa*. In turn, however, *P. aeruginosa* forms a dense biofilm on *C. albicans* hyphae and kills the fungus [21]. *Candida* colonization of the respiratory tract was also shown to be common in patients receiving mechanical ventilation and was associated with prolonged ICU and hospital stays, and with an increased risk of *Pseudomonas* ventilator-associated pneumonia [22]. The clinical significance of the high levels of coexistence of *P. aeruginosa* and *Candida* spp. that were observed in the present study and their interaction with each other in patients with CF remains to be studied.

Both methicillin-susceptible *S. aureus* (MSSA) and methicillin-resistant *S. aureus* (MRSA) remain important pathogens both early and late in the disease course of CF [23]. Colonization by MRSA has been most strongly associated with lower lung function in CF patients [20]. The interaction between *S. aureus* and *C. albicans*, although not yet fully characterized, appears to be initially synergistic [24]. In the present study, we showed that CF patients had higher levels of *S. aureus* with *Candida* spp. than patients with other respiratory disorders, 13.1% vs. 5.9%, respectively (*p* < 0.05). It is important to note that none of the *S. aureus* isolates detected in this study were MRSA probably due to its very low prevalence in Sweden.

A majority of the recent studies on this subject have analyzed the microbiota at the molecular level. The major limitation of culture-based methods is their inability to detect non-culturable microorganisms. However, culture-based methods are reference methods in clinical routine for the detection of CF pathogens in the airways and enable the detection of viable microorganisms. In addition, molecular-based methods including next generation sequencing detect even low amounts of microorganisms, whereas the detection by culture-based methods requires high levels of microorganisms existing in the sample which probably reflects the clinical relevance of individual bacteria and yeast better.

It is important to analyze the coexistence of different microorganisms in individual chronic diseases. However, in the absence of comparison with other diseases, the interpretation of the data is inevitably limited. The present study is one of the first studies analyzing the coexistence of pathogenic bacteria and *Candida* spp. in patients with CF and comparing the data with a control group. The high existence of yeasts in the absence of high coexistence of pathogenic bacteria and yeasts in the control group indicates that colonization by *Candida* spp. does not exclusively lead to higher existence of pathogenic bacteria.

In general, it is difficult to find the optimal control group for CF patients. In the present study, patients with respiratory disorders other than CF were used as the control group. One of the limitations with this control group was that the controls were not age-matched with the CF patient group. However, the patients in the control group were from different lung and allergy units, suggesting that the patients had chronic respiratory tract diseases and may be compared with CF patients.

In conclusion, the present data show that CF is related to high levels of coexistence of pathogenic bacteria and *Candida* spp. Further studies are warranted to analyze the underlying reasons and the clinical relevance of the coexistence of pathogenic bacteria and *Candida* spp. described in the present study to guide relevant antifungal and antibacterial treatments for patients with CF.
